# Supplementary material for: Uncovering overlooked bacterial infections in gene X-pert MTB/RIF negative sputum samples of adult patients with presumptive tuberculosis in Harar, Eastern Ethiopia
Source: PLoS One. 2026 May 14;21(5):e0348978. doi: 10.1371/journal.pone.0348978 (PMC13175332; doi:10.1371/journal.pone.0348978)
Supplement: Attachment — Submitted filename: PONE-D-25-64253 reviewed.pdf [file pone.0348978.s002.pdf]

PLOS One

Uncovering Overlooked Bacterial Infections in GeneXpert MTB/RIF-Negative Sputum Samples of adult patients with presumptive tuberculosis in Harar, Eastern Ethiopia

--Manuscript Draft--

**Manuscript Number:**	PONE-D-25-64253
**Article Type:**	Research Article
**Full Title:**	Uncovering Overlooked Bacterial Infections in GeneXpert MTB/RIF-Negative Sputum Samples of adult patients with presumptive tuberculosis in Harar, Eastern Ethiopia
**Short Title:**	Uncovering overlooked Bacterial Infections in GeneXpert MTB/RIF-negative sputum samples
**Corresponding Author:**	Mohammed Ahmed, MSc<br>Haramaya University College of Health and Medical Sciences<br>Harar, Harari ETHIOPIA
**Keywords:**	Bacterial pathogens, Antimicrobial resistance; Xpert-negative presumptive tuberculosis; Eastern Ethiopia
**Abstract:**	Background<br><br>Lower respiratory tract infections are one of the diseases that pose an existential threat to global health. Clinical presentations of various bacterial lower respiratory infections and presumptive pulmonary tuberculosis are often overlap, hindering prompt patient care and correct diagnosis. In developing countries like Ethiopia, the management of presumptive tuberculosis cases that test negative for Gene Xpert are often empirical without determining the underlying causes of the illness, which leads to treatment delays and the development of antibiotic resistance. Therefore, it is imperative to address potential overlooked bacterial infections in cases that are negative for tuberculosis.<br><br>Objective<br><br>This study aimed to determine the prevalence of pathogenic bacteria, antimicrobial susceptibility patterns, and associated factors of lower respiratory tract infection among Xpert-negative presumptive tuberculosis adult patients at Hiwot Fana Comprehensive Specialized Hospital, eastern Ethiopia.<br><br>Method<br><br>A hospital-based cross-sectional study was conducted on 371 study participants from January to April 2024. Socio-demographic and health related data were collected using structured questionnaire. Gene Xpert MTB/RIF was used for initial tuberculosis screening, and sputum culture was used to isolate bacterial pathogens. Bacterial isolates were identified based on gram staining, colony characteristics, and biochemical reactions. Antimicrobial susceptibility test was done using Kirby-Bauer disc diffusion method. Methicillin resistance S. aureus was confirmed using cefoxitin (30 µg). Logistic regression analysis was used to assess the association between outcome and predictor variables.<br><br>Results<br><br>The overall prevalence of pathogenic bacterial lower respiratory tract infection was 34.0% (95% CI; 29.2, 38.8). Gram-negative bacteria accounted for 66.7%. S. aureus (19.0%) was the predominant isolate followed by K. pneumonia (14.3%). The proportion of multi-drug resistant bacteria and methicillin-resistance S. aureus was 39.7% and 16.7% respectively. Primary education (AOR=4.01; 95% CI=1.62, 9.92), history of antibiotic usage (AOR=1.87; 95% CI=1.13, 3.08) and crowded living condition (AOR=5.11; 95% CI=3.03, 8.61) were factors associated with pathogenic bacterial lower respiratory tract infections.<br><br>Conclusion

Powered by Editorial Manager® and ProduXion Manager® from Aries Systems Corporation

	This study revealed that patients with presumptive tuberculosis who tested negative on gene Xpert- were predominantly infected with gram negative pathogenic bacteria. Therefore, it's important to prioritize sputum culture and antibiotic susceptibility testing. This study underscore the need to avoid themisuse of antibiotics and crowded living conditions.
**Order of Authors:**	Negesso Gebre, MSc
	Kedir Urgesa, PhD
	Jemal Mohammed, MSc
	Rudwan Yasin Abrahim, BSc degree in Medical doctor
	Mohammed Ahmed, MSc
**Additional Information:**	
**Question**	**Response**
**Financial Disclosure**<br><br>Enter a financial disclosure statement that describes the sources of funding for the work included in this submission. Review the submission guidelines for detailed requirements. View published research articles from *PLOS ONE* for specific examples.<br><br>This statement is required for submission and **will appear in the published article** if the submission is accepted. Please make sure it is accurate.<br><br>**Funded studies**<br>Enter a statement with the following details:<br>• Initials of the authors who received each award<br>• Grant numbers awarded to each author<br>• The full name of each funder<br>• URL of each funder website<br>• Did the sponsors or funders play any role in the study design, data collection and analysis, decision to publish, or preparation of the manuscript?<br><br>Did you receive funding for this work?	The author(s) received no specific funding for this work.
**Competing Interests**<br><br>Use the instructions below to enter a competing interest statement for this submission. On behalf of all authors, disclose any competing interests that	The authors have declared that no competing interest exists

could be perceived to bias this work—acknowledging all financial support and any other relevant financial or non-financial competing interests.<br><br>This statement is required for submission and **will appear in the published article** if the submission is accepted. Please make sure it is accurate and that any funding sources listed in your Funding Information later in the submission form are also declared in your Financial Disclosure statement.<br><br>View published research articles from *PLOS ONE* for specific examples.<br><br>**NO authors have competing interests**<br><br>Enter: *The authors have declared that no competing interests exist.*<br><br>**Authors with competing interests**<br><br>Enter competing interest details beginning with this statement:<br><br>*I have read the journal's policy and the authors of this manuscript have the following competing interests: [insert competing interests here]*<br><br>* typeset	
**Ethics Statement**<br><br>Enter an ethics statement for this submission. This statement is required if the study involved:<br><br>• Human participants<br>• Human specimens or tissue<br>• Vertebrate animals or cephalopods<br>• Vertebrate embryos or tissues<br>• Field research<br><br>Write "N/A" if the submission does not require an ethics statement.	Ethical clearance was obtained from Haramaya University, College of Health and Medical Sciences, through the Institutional Health Research Ethics Review Committee (IHRERC) (Ref. No. IHRERC/159/2023). A permission letter to conduct this research was obtained from Hiwot Fana Comprehensive Specialised University Hospital. Study subjects were given information about the study's objectives and provided written informed consent or a thumbprint for subjects who were unable to write.

Powered by Editorial Manager® and ProduXion Manager® from Aries Systems Corporation

General guidance is provided below. Consult the submission guidelines for detailed instructions. **Make sure that all information entered here is included in the Methods section of the manuscript.**<br><br>**Format for specific study types**<br><br>**Human Subject Research (involving human participants and/or tissue)**<br>• Give the name of the institutional review board or ethics committee that approved the study<br>• Include the approval number and/or a statement indicating approval of this research<br>• Indicate the form of consent obtained (written/oral) or the reason that consent was not obtained (e.g. the data were analyzed anonymously)<br><br>**Animal Research (involving vertebrate animals, embryos or tissues)**<br>• Provide the name of the Institutional Animal Care and Use Committee (IACUC) or other relevant ethics board that reviewed the study protocol, and indicate whether they approved this research or granted a formal waiver of ethical approval<br>• Include an approval number if one was obtained<br>• If the study involved *non-human primates*, add *additional details* about animal welfare and steps taken to ameliorate suffering<br>• If anesthesia, euthanasia, or any kind of animal sacrifice is part of the study, include briefly which substances and/or methods were applied<br><br>**Field Research**<br><br>Include the following details if this study involves the collection of plant, animal, or other materials from a natural setting:<br>• Field permit number<br>• Name of the institution or relevant body that granted permission	
**Data Availability**<br><br>Authors are required to make all data	Yes - all data are fully available without restriction

Powered by Editorial Manager® and ProduXion Manager® from Aries Systems Corporation

underlying the findings described fully available, without restriction, and from the time of publication. PLOS allows rare exceptions to address legal and ethical concerns. See the PLOS Data Policy and FAQ for detailed information.<br><br>A Data Availability Statement describing where the data can be found is required at submission. Your answers to this question constitute the Data Availability Statement and **will be published in the article**, if accepted.<br><br>**Important:** Stating 'data available on request from the author' is not sufficient. If your data are only available upon request, select 'No' for the first question and explain your exceptional situation in the text box.<br><br>Do the authors confirm that all data underlying the findings described in their manuscript are fully available without restriction?	
**Describe where the data may be found in full sentences. If you are copying our sample text, replace any instances of XXX with the appropriate details.**<br><br>• If the data are **held or will be held in a public repository**, include URLs, accession numbers or DOIs. If this information will only be available after acceptance, indicate this by ticking the box below. For example: *All XXX files are available from the XXX database (accession number(s) XXX, XXX.*).<br>• If the data are all contained **within the manuscript and/or Supporting Information files**, enter the following: *All relevant data are within the manuscript and its Supporting Information files.*<br>• If neither of these applies but you are able to provide **details of access elsewhere**, with or without limitations, please do so. For example:<br><br>*Data cannot be shared publicly because of [XXX]. Data are available from the*	The data underlying the results presented in the study are available from the corresponding author and the first author

XXX Institutional Data Access / Ethics Committee (contact via XXX) for researchers who meet the criteria for access to confidential data.<br><br>*The data underlying the results presented in the study are available from (include the name of the third party and contact information or URL).*<br>• This text is appropriate if the data are owned by a third party and authors do not have permission to share the data.<br><br>* typeset	
Additional data availability information:	

Powered by Editorial Manager® and ProduXion Manager® from Aries Systems Corporation

Manuscript
Click here to access/download;Manuscript;Manuscript LRTIs among_Xpert -ve presumptive TB.docx

Uncovering Overlooked bacterial Infections in Gene X-pert MTB/RIF negative sputum samples of adult patients with presumptive tuberculosis in Harar, Eastern Ethiopia

Negesso Gebre[1], Kedir Urgesa[2], Jemal Mohammed[2], Rudwan Yasin Abrahim[3], Mohammed Ahmed[2*]

Authors' Affiliations

[1] Student higher Clinic, Haramaya University, Haramaya, Oromia, Ethiopia

[2] School of Medical Laboratory Sciences, College of Health and Medical Sciences, Haramaya University, Harar, Ethiopia

[3]School of Medicine, College of Health and Medical Sciences, Haramaya University, Harar, Ethiopia

***Corresponding Authors**

Email: mameahmed129@gmail.com**(MA)**

ORCID: *https://orcid.org/0000-0002-4999-8137*

ABSTRACT

Background: Lower respiratory tract infections are one of the diseases that pose an existential threat to global health. Clinical presentations of various bacterial lower respiratory infections and presumptive pulmonary tuberculosis are often overlap, hindering prompt patient care and correct diagnosis. In developing countries like Ethiopia, the management of presumptive tuberculosis cases that test negative for Gene Xpert are often empirical without determining the underlying causes of the illness, which leads to treatment delays and the development of antibiotic resistance. Therefore, it is imperative to address potential overlooked bacterial infections in cases that are negative for tuberculosis.

Objective: This study aimed to determine the prevalence of pathogenic bacteria, antimicrobial susceptibility patterns, and associated factors of lower respiratory tract infection among Xpert-negative presumptive tuberculosis adult patients at Hiwot Fana Comprehensive Specialized Hospital, eastern Ethiopia.

Method: A hospital-based cross-sectional study was conducted on 371 study participants from January to April 2024. Socio-demographic and health related data were collected using structured questionnaire. Gene Xpert MTB/RIF was used for initial tuberculosis screening, and sputum culture was used to isolate bacterial pathogens. Bacterial isolates were identified based on gram staining, colony characteristics, and biochemical reactions. Antimicrobial susceptibility test was done using Kirby-Bauer disc diffusion method. Methicillin resistance *S. aureus* was confirmed using cefoxitin (30 µg). Logistic regression analysis was used to assess the association between outcome and predictor variables.

Results: The overall prevalence of pathogenic bacterial lower respiratory tract infection was 34.0 % (95% CI; 29.2, 38.8). Gram-negative bacteria accounted for 66.7%. *S. aureus* (19.0 %) was the predominant isolate followed by *K. pneumonia* (14.3%). The proportion of multi-drug resistant bacteria and methicillin-resistance *S. aureus* was 39.7% and 16.7% respectively. Primary education (AOR=4.01; 95% CI=1.62, 9.92), history of antibiotic usage (AOR=1.87; 95% CI=1.13, 3.08) and crowded living condition (AOR=5.11; 95% CI=3.03, 8.61) were factors associated with pathogenic bacterial lower respiratory tract infections.

Conclusion: This study revealed that patients with presumptive tuberculosis who tested negative on gene Xpert- were predominantly infected with gram negative pathogenic bacteria.

Therefore, it's important to prioritize sputum culture and antibiotic susceptibility testing. This study underscore the need to avoid themisuse of antibiotics and crowded living conditions.

Keywords: Bacterial pathogens, Antimicrobial resistance; Xpert-negative presumptive tuberculosis; eastern Ethiopia

Introduction

Globally, lower respiratory tract infections (LRTIs) are a leading causes of morbidity and mortality, particularly in developing countries [1, 2]. Global burden of disease study 2019 indicates that, in the last three decade LRTIs cause an estimated 489 million incidence cases and 2.4 million deaths of which Sab-Saharan Africa regions accounts largest incidence and death from lower respiratory infections [3].In Ethiopia, LRTIs are the main reason for hospital admissions and the third leading cause of death, accounting 8.2% of all deaths reported in 2019 [4]. In Xpert negative presumptive tuberculosis common bacteria that causes LRTIs are *k .pneumonia*, *S. aureus*, *H. influenza* and *S. pneumonia*[5, 6].

According to the endorsement by WHO (2017), the Xpert MTB/RIF assay is highly specific (98%) to give an excellent performance for the diagnosis of TB [7]. Thus, patients with gene-Xpert MTB/RIF negative are unlikely to suffer from pulmonary tuberculosis [8]. However, LRTIs that have similar manifestations with TB are poly-microbial (bacteria, fungi, viruses, and parasites) in nature and rendering emergency of Antimicrobial resistance (AMR) [9, 10].

Antimicrobial resistance (AMR) by pathogenic bacteria is dramatically accelerated by exploiting antibiotics in the communities and health facilities, leading to the emergence of resistant bacteria [11]. Methicillin Resistant *Staphylococcus aureus* (MRSA) is one of as the emerging multi-drug resistance strain circulating in health facilities and communities [12, 13]. Bacterial LRTI pathogens are associated with different factors including old age, crowded living conditions, smoking, comorbidities, and history of prior antibiotic usage [14, 15].

According to Ethiopian national guidelines for management of TB, DR-TB and leprosy by Ethiopian Federal Ministry of Health (2018), patients with respiratory symptoms with whom tuberculosis (TB) is less likely are empirical [16]. However, when gene Xpert MTB/RIF negative presumptive pulmonary TB, cases are predominantly infected with other pathogenic bacteria than TB [17], which could result in poor patient’s prognosis and prone to emergence of multi-drug resistant bacterial pathogens [18]. Hence, in the case of TB diagnosis rolled out,

considering the other pathogenic bacterial etiology of LRTIs is important for accurate diagnosis and optimal patient management [19].

Although few studies have been done in Ethiopia on bacterial LRTIs ([20-22], study on the pathogenic bacteria lower respiratory infection, antimicrobial susceptibility patterns and associated factors among presumptive tuberculosis adult patients with gene Xpert-negative is rare. Thus, this study aimed to determine the prevalence of pathogenic bacteria, antimicrobial susceptibility patterns and associated factors of lower respiratory tract infections among Xpert-negative presumptive pulmonary tuberculosis adult patients at Hiwot Fana Comprehensive Specialized Hospital, Harar, eastern Ethiopia.

Additional study is needed to understand the impacts of bacterial LRTIs on patient's outcomes, antibiotic resistance pattern and better patient managements.

Materials and methods

Study area and period

The study was conducted at Hiwot Fana Comprehensive Specialized Hospital (HFCSH) in Harar, Harari region, eastern Ethiopia from January 01 to April 30, 2024Harar is a capital of the region located 525 km away from the capital city of Addis Ababa. The hospital serves more than 5 million people in the Harari Region, Eastern Hararghe, and West Hararghe, Dire Dawa and Somali regions and clients from neighboring countries. According to HFCSH, Health Management Information System (HMIS) report (2023), the hospital has more than 34 outpatient department, 15 inpatient departments and a separate emergency and trauma center, and serves as the only cancer treatment center in Eastern Ethiopia. The hospital offers full laboratory services to the public. The Xpert MTB/RIF assay is one of the laboratory services offered to patients with signs and symptoms of presumptive tuberculosis. According to the annual report of hospital service, 3500 was identified as a presumptive TB cases.

Study design, Population, Sampling technique and Procedure

An institutional-based cross-sectional study was conducted. Presumptive pulmonary tuberculosis patients from both outpatient and inpatient department who visited laboratory for Gene Xpert MTB/RIF assay services and adult patients aged ≥ 18 years with Xpert-negative who consented were included in the study. Convenient sampling method were used to select participantsuntil the calculated sample was fulfilled.

The Adult patients who received antibiotic treatments within two weeks prior to study period, severely ill patients and those who could not provide sputum samples were excluded from the study.

Sample size determination

The sample size was determined using a single population proportion formula by taking the prevalence (33.5%) from a previous study done in Hawassa [20]. Considering the 95 % confidence interval CI (z=1.96), margin error (5%), (d = 0.05). The calculated sample size was 342 Therefore, final sample size was 376 after adding 10% non-respondents.

Operational definition

Lower respiratory tract infections are defined as any infections in the lower respiratory tract with common symptoms like cough, fever, shortness of breath, chest pain or tightness, which include pneumonia, bronchitis, and bronchiolitis [2].

Xpert MTB/RIF-negative presumptive pulmonary TB cases: refers to cases where Gene Xpert MTB/RIF assay does not detect mycobacterium tuberculosis and rifampicin resistance in patients suspected with presumptive pulmonary TB [5].

Crowded living condition: refers to if there are more than three people per habitable room or more than two people per bedroom [23].

Data collection methods

Data were collected by two trained professional nurses and one medical laboratory technologist. All relevant data, including socio-demographic variables, behavioral factors, health-related conditions, and environmental factors, was collected using a pre-tested structured questionnaire adapted from different literatures [5, 17, 19, 20, 24, 25].

Sputum samples collection, and Bacterial identification method

Purulent sputum sample were collected into a wide mouthed screw plastic cup from all participants. Prior to sputum collection participants were briefly instructed to rinse their mouth with water and take deep breath to expectorate purulent sputum. The quality of collected sputum samples were assessed using Bartlett's criteria through the score of pus cells, and squamous epithelial cells on gram stain. Based on gram stain, the sputum samples with greater than 25 polymorphonuclear and less than 10 epithelial cells per low power field were accepted for culture and transported immediately to microbiology laboratory within less than 2 hours of collection.

Then, Sputum samples were inoculated onto Blood agar (Oxoid Ltd., Basingstoke, UK), Chocolate agar (Oxoid Ltd., Basingstoke, UK) and MacConkey agar (Oxoid Ltd., Basingstoke, UK) by using sterile wire loop. Briefly, MacConkey agar (Oxoid Ltd., Basingstoke, UK) plates were incubated aerobically at 37 ^{o}C for 24 hours, whereas Blood and Chocolate agar plates were incubated within 5% CO_2 generating candle jar at 37 ^{o}C for 24 hours. The plates were inspected for the growth after 24 hours of incubations. The pure isolates of bacterial pathogens were characterized by colony morphology, gram stain, and hemolytic reaction. Identification of pathogenic bacteria down to species level were done by using biochemical tests such as Catalase, Coagulase, Bacitracin, Optochin , and bile solubility for identification of gram-positive bacteria, whereas IMViC, TSI, Oxidase test and Urease tests were used for identification of gram-negative bacteria [26].

Antimicrobial susceptibility testing

Antimicrobial susceptibility test (AST) was performed using a modified Kirby-Bauer disk diffusion technique as recommended by Clinical Laboratory Standard Institute Guideline stated in CLSI (2022). Suspension of isolated bacteria was done by using normal saline 0.9% to emulsify colonies until matching to 0.5% McFarland standard solution. Using a sterile cotton swab, sufficient inoculum was distributed on Muller-Hinton agar plates with or without 5% sheep blood with (Oxoid Ltd., Basingstoke, UK). *H. influenzae* media (HIM) was used to perform AST for H. *influenza* as stated in *CLSI* (2022). After 5 minutes, a set of selected antimicrobial disks were aseptically placed on Mueller-Hinton agar plates and allowed to stand at room temperature for 15 minutes. Then, all plates were incubated at 37^0C for 24 hours while maintaining all the requirements of the respective bacteria as done during the isolation process. The diameters of the zone of inhibition around the disk was measured using a ruler and compared to reference points stated in CLSI (2022) and interpreted as sensitive (S), intermediate (I), and resistant (R) [27]. Isolates were classified as either susceptible or resistant to an antibiotic and all isolates with intermediate resistance were classified as resistant for a better fit in further statistical analysis. Multi-drug resistance (MDR) in this study was extrapolated as resistance to three or more groups of antibiotics tested [28]. Isolates of Methicillin *S. aureus (MRSA)* were detected according to the CLSI guidelines by using cefoxitin disc. Fifteen antibiotics were selected based on CLSI (2022) recommendation, guideline and routine local antibiotic usage, which include Ampicillin (10µg), Amoxicillin-clavunate (30µg), Penicillin (10µg), Piperacillin/tazobactam(100µg), Ceftriaxone(30µg), Ceftazidime (30 µg), Cefoxitin (30 µg), Gentamicin (10 µg), Erythromycin (15 µg), Azithromycin(15µg), Cotrimoxazole) (12µg),Tetracycline (30µg),clindamycin (2µg), Ciprofloxacin(5µg) and Meropenem (20 µg).

Data quality control

Prior to the actual data two days training was given for data collectors by the principal investigator on the data collection tools and the sample collection procedures. The questionnaire was pre-tested on 5% of gene Xpert–negative presumptive tuberculosis adult patients in Jugal General Hospital to check the validity and the applicability of the questionnaire. All Sputum specimens were collected and processed according to the Standard operating procedure (SOP).

The quality of the specimen was checked based on Bartlett's criteria (Popova et al., 2019). The sterility of the culture media was ensured by incubating 5% of each batch of the prepared media at 37°C for 24 hrs. To standardize the inoculum density of bacterial suspension, turbidity was adjusted using 0.5 McFarland standard [26]. The performance of all prepared media was checked by inoculating standard ATCC strains such as *S. aureus* (ATCC -25923) and *E.coli* (ATCC-25922) (CLSI, 2022).

Data analysis

Data were entered into EPI-Data version 4.60 and analyzed using Statistical Package for Social Science (SPSS) program version 20. Results were presented by using text, Table, and figure.. Variables with a p-value ≤ 0.25 in bi-variable analysis were selected candidates for multivariable analysis. Finally, the multivariable logistic regression analysis were used for final decisions with odds ratio and 95% confidence intervals. Statistical significance was declared at a p-value < 0.05.

Results

Socio-demographic characteristics of the study participants

From the total of 376 Xpert MTB/RIF-negative adult patients presenting with symptoms of pulmonary TB, 371 were enrolled with a response rate of 98.7 %. The proportion of male patients was 197/371 (53.1%). The mean age (±SD) of study participants was 38 ± 15.69. Rural residents accounts 213/371 (57.4%), farmers accounted 129/371 (34.8%), and 146/371 (39.4%) of participants have no formal education (Table 1).

Clinical, Behavioral, and Environmental characteristics of the study participants

More than half of study participants 192 (51.8%) had a history of antibiotic usage for a month or more before the study period. More than ten percent 40(10.8%) of participants had a previous history of hospital admission whereas 17(4.6%) of them had a previous history of tuberculosis.

From the total study participants, thirty percent 113(30.5%) of them had the habit of smoking cigarettes, and 155 (41.8%) of them lived in crowded conditions. Majority of them used firewood as a source of energy for cooking 226(60.9%) and half of them 187(50.4%) lived in a mud/soil floor house (Table 2).

Prevalence of bacterial LRTI pathogens

The overall prevalence of bacterial LRTIs pathogens was 34.0 %, (95% CI; 29.2, 38.8). Out of 126 isolated bacterial pathogens, two-third of them were from Gram-negative bacteria (GNB) 84/126(67%) whereas one-third of them corresponded to Gram-positive bacteria 42/126(33%) Among GNB isolates, *Klebsella species* accounted highest percentage. These includes *K. pneumoniae* 18/126(14.3%), *K. oxytoca* 10/126(7.9%), *K. ozanae* 9/126(7%), and *K. rhinoscleromatis* 13/126(10.3%). The prevalence of other GNB isolates were *E. coli* 8/126(6.3%), *Entrobacter species* 7/126(5.6%), *Citrobacter species* 4/126(3.2%), *Proteus mirabilis* 1/126(0.8%), *H. influanzae* 6/126(4.8%) and *M. catarhallis* 8/126(6.3%). The isolates from Gram-positive bacteria category revealed three isolates namely: *S. aurues* 24/126(19%), *S. pneumonia* 13/126(10.3%), and *S. pyogens* 5/126(4%). *S. aureus* (19.0%) showed the highest predominance among all isolates followed by *K. peumoniae*, (14.3%). *K. rhinoscleromatis* (10.3%) *and S. pneumonia* (10.3%) *showed* similar rate of distribution which put them in third place (Figure 1).

Antimicrobial susceptibility patterns of the bacterial isolates

In this study, the antimicrobial susceptibility patterns of Gram-positive bacteria (n=42) =isolates produced wider variations in their susceptibility patterns. Overall, gram-positive isolates were susceptible to clindamycin 29/42(69.05%) and erythromycin 25/42(59.5%) while 31/42 (73.8%) was resistant to tetracycline Isolates of *S. aureus* were resistant to penicillin 19/24 (79.2%), trimethoprim–sulfamethoxazole 14/24(58.3%) however, 20/25(83.3%) isolates were susceptible to (cefoxitin, ciprofloxacin, and gentamycin), and 18/24(75%). azithromycin *Among isolates of S. aureus* 4/24 (16.7%) showed zone of inhibition ≤21 mm (16.2 to 19.5 mm)), in the cefoxitin disc diffusion assay and they were identified as methicillin-resistant (MRSA). The isolates of *S. pneumonia* were resistant to erythromycin 7/13(53.8%), tetracycline 7/13(53.8%),

and sensitive to clindamycin 10/13(76.9%), and co-trimoxazole 8/13(61.5%). The isolates of *S. pyogenes* were resistant to clindamycin 2/5(40%), erythromycin 3/5(60%), However, all isolates of *S. pyogenes* showed susceptibility towards tetracycline (Table 3).

The antimicrobial Susceptibility patterns of Gram-negative bacteria (n=84) isolates against nine antibiotics were as follows,almost all of the Gram-negative isolates were susceptible to piperacillin/tazobactum 81/84(96.4%), meropenem 82/84(97.6%); and resistant to ceftriaxone 24/84 (28.6%), and gentamycin 23/78 (29.5%) respectively. The isolates of *K.peumonia* were found to be resistant to gentamycin, 7/18 (38.9%), amoxicillin-clavulanate (augmentin), 12/18 (66.7%), while all isolates were highly susceptible to piperacillin/tazobuctum, and meropenem. The majority of *E. coli* isolates were resistant to ampicillin, 7/8(88.5%), amoxicillin-clavulanate, 5/8(62.5%) but all isolates were susceptible to piperacillin, and meropenem. A single isolated of *P.mirabilis* was susceptible to all set of used antibiotics. All isolates of *H.influanzae* were resistant to ampicillin but all of them were susceptible to ciprofloxacin, piperacillin, and meropenem. The isolates of *M. catarrhalis* were highly resistant to co-trimoxazole, but all isolates were susceptible to ceftazidime, piperacillin, and meropenem (Table 4).

Multi-drug resistance patterns of bacterial isolates

Multi-drug resistance (MDR) was found in 50/126 (39.7 %). About a third of isolates 16/50 (32%) were resistant to 3 antibiotics and 21/50 (42%) of them were resistant to five and more antibiotics. Multi-drug resistance pattern (MDR) was accounted 35/84 (41.7%) in Gram-negative bacteria isolates with *E. coli* 6/8 (75%) the predominant MDR species, with followed by *M. catarrhalis* 4/8 (50%), *K. rhinoscleromatis* 6/13 (46.2%) whereas *S. pyogens* 2/5 (40%) were the dominant MDR species among gram positive isolates. The least percentage of MDR was shown in the isolates of *H. influenza 1/6* (16.7%) (Table 5).

Factors associated with bacterial LRTI pathogens

In the bi-variable analysis, gender, educational status, history of hospital admission, history of antibiotic usage prior a month and above, habit of hand washing after outing, crowded living conditions, separate kitchen from house, living in mud floor, and source of drinking water were significantly associated with bacterial LRTI pathogens with *p*-value less than 0.25. However, in

the multivariable logistic regression analysis model, educational status, history of prior antibiotic usage and crowded living conditions remained associated with bacterial LRTI pathogens at *p*-value less than 0.05.

Study participants who had primary educational status were four times (AOR=4.008; 95% CI=1.619, 9.920; *p* =0.003) more likely to be infected with bacterial LRTI pathogens compared to those who had college and above educational status. Study participants who had a history of antibiotic usage prior a month and above) were two times (AOR=1.866; 95% CI=1.132, 3.077; *p* =0.014) more likely to be infected with bacterial LRTI pathogens compared to their counterparts. Study participants who were living in a crowded conditions were five times (AOR=5.106; 95% CI=3.028, 8.611; *p* <0.001) more likely to be infected with bacterial LRTI pathogens compared to counterparts (Table 6).

Discussion

Lower respiratory tract infections (LRIs) are a significant public health problem and a leading cause of morbidity and mortality in persons of any age[15]. In the current study, the overall prevalence of bacterial LRTIs among Xpert-negative presumptive pulmonary tuberculosis adult patients was found to be 34.0% (95% CI; 29.2, 38.8). Comparable findings were reported from previous studies conducted in Hawassa (33.5%) [20], and Addis Ababa, Ethiopia [21]. However, this prevalence was lower than the study conducted in couple of cities in Ethiopia Gondar(57.1%) [5] and Jimma (80%) [19], Cameroon (46.8%) [29], Cambodia (44%) [30], Nepal (47.33%) [25], Philippines (90.24%) [31]. This discrepancy might be due to variation in diagnosis methods used by those studies that increased detection capacity compared to ours. They used molecular test like commercial reverse-transcriptase real-time polymerase chain reaction (RT-PCR) assay and 16S rRNA gene amplicon sequencing was used by aforementioned studies which results in an increasing the detection rate of all bacteria than conventional method we used [29, 31, 32]. However, this study is higher than study conducted in Tanzania(16.4%) [17]. This variation could be due to geographical variation, difference in study population and sample size, [33].

All culture positive rate of bacteriological confirmed cases were found to be mono-bacterial infections. This study is consistent with the study reported from Ghana [34]. However, it is different from studies done in different parts of Ethiopia, Addis Ababa [21] , Gondar [5], Hawassa [20], Tanzania [17], Iran [35] and China [36]. The difference might be due to the difference in used empirical treatmentsm variation of diagnosis method, and sample size.

The most common pathogenic bacterial isolatewere *S. aureus, S. pneumonia, S. pyogens*, *K. penumoniae , K. oxytoca, K.ozanae, K. rhinoscleromatis, Enterobacter species, Citrobacter species, E. coli , H. influnzae , M. catarhallis, and P. mirabillis*. This is concordance with the study done in Gondar, Ethiopia [5] and Tanzania [17]. However, this study was inconsistent with studies done in Nepal [25] and Philippines [31]. This might be due to that the etiologic agents may vary with epidemiological variation [11, 29]. The two-third of bacterial isolates were belongs to gram-negative bacteria 84 (66.7%) and one-third of Gram-positive bacteria correspond to 42 (33.3%). The same trend were reported bystudy done in several parts of

Ethiopia ,Addis Ababa(77.8%) [21], Gondar (64.9%) [5], and Hawassa (76.1%) [20] and other countries like Tanzania (75%) [17] and China (72.6%) [36]. This might be due to that GNB have strong pathogenic ability and virulence factors such as Type1 pili, which may enhance the ability to adhere and colonize the lower respiratory tract and outer membrane that aid in pathogenesis and immune evasions [37]. However, this is in contrast to studies reported in Iran [35], Nigeria [38], and Jimma, Ethiopia [39] that Gram-positive bacteria were as the dominant group. *S. aureus* 24 (19%) was categorized to gram-positive bacteria and the most dominant isolates among all. Similar dominance of *S. aureus* isolates was noted in the studies carried out in different places, Gondar Ethiopia [5], and in Pakistan [40].

The high prevalence of S. aureus may be attributed to its extensive presence in both hospital and community environments. Furthermore, *S. aureus* persistence in respiratory tract is supported by a variety of traits including demonstrating metabolic versatility, its capacity to scavenge iron, coordinated regulation gene expression, horizontal genes transfer, and expression of surface adhesions[41].

K. pneumonia (14.3%) were the second most frequently isolated bacteria. This is in line with study carried out in Gondar, Ethiopia [5] and Tanzania [17]. Most of Gram-positive bacteria (83%) including *S. aureus* isolates were highly susceptible to ciprofloxacin, gentamicin, and cefoxitin while showed resistance to penicillin (79.2%) and tetracycline (73.8%). These observations are in line with several studies done across different parts of Ethiopia [5, 20, 21, 36]. It is to be noted that almost all of the isolates of Gram-negative bacteria were highly susceptible to piperacillin/tazobactam (96.4%) and meropenem (97.6%) respectively while exhibiting resistance to amoxicillin-clavulanate. This is inline with the study carried out in Addis Ababa, Ethiopia [21]. All isolates of *K. pneumonia* were susceptible to piperacillin/tazobactam (100%) and meropenem (100% and on the contrary half of them were resistant to trimethoprim–sulfamethoxazole (50%) whereas two-third of them were resistant to amoxicillin-clavulanate (66.7%). These are comparable to the results of past studies done in a couple of cities of Ethiopia [20, 21] and China [36].

In this study, MDR was observed in the case of 50 (39.7%) of isolates, which is comparable to a study conducted in Hawassa, Ethiopia (32.4%) [20]. However, lower than studies done in Addis Ababa, Ethiopia (80.2%) [21] and Nepal (55.56%) [25]. The difference of antibiotic

resistance may be owing to wide spread irrational use and over exploitation of antimicrobial agents [42]. Among the MDR, Majority were corresponding to Gram-negative bacteria 35 (41.7%). These findings are in line with the results of a study conducted in Nepal [25]. The might be due to GNB increasing the intrinsic resistance (mutations) in chromosomal genes (such as increasing the expression of antibiotic-inactivating enzymes, efflux pumps, permeability or target modifications) or acquired by transfer of mobile genetic elements carrying resistance genes such as plasmid [43].

In this study, the methicillin resistant *S. aureus* (MRSA) strains were found to be 4 (16.7%). A similar pattern of resistance was observed in a study done in Tanzania [17]. This might be due to that MRSA circulating in in health facilities and communities from frequent use of broad antibiotics leads to spread of strain that produces beta-lactamase, and through expression of mecA gene that encode production of an unusual penicillin-binding protein and modification of penicillin-binding protein results in weakening the affinity for β-lactam antibiotics[44, 45].

Among the various factors assessed contributing to bacterial LRTI pathogens, primary education, history of prior antibiotic usage and crowded living conditions were found to be significantly associated with bacterial LRTI pathogens. Study participants who had primary educational status were 4.008 times (AOR=4.008; 95% CI=1.619, 9.920; p =0.003) more likely to be infected with bacterial LRTI pathogens compared to those who had college and above educational status. It is in line with studies done in Wolaita Sodo, Ethiopia [46] and China [47]. This may be due to the fact that low education could be a good proxy for conditions of vulnerability because of diminished awareness or on another way high levels of education would allow us a better understanding about preventive measures such as vaccination [48] . Study participants who had a history of antibiotic usage prior a month and above) were 1.866 times (AOR=1.866; 95% CI=1.132, 3.077; p =0.014) more likely to be infected with bacterial LRTI pathogens compared to counterparts. It is consonant with a couple of studies conducted in China [36, 49] and USA [50]. The probable reason for this could be due to antibiotic resistance in the bacteria following the frequent exposure to antibiotics [42, 51]. Study participants who were living in a crowded condition were 5 times (AOR=5.106; 95% CI=3.028, 8.611; p <0.001) more likely to be infected with bacterial LRTI pathogens compared to counterparts. The similar patterns showed in a couple of studies done in Gondar and Felege Hiwot, Ethiopia [24, 52]. This

might be due to the fact that crowdedness facilitates the proximity of individuals to each other to increase the possibility of aerosol transmission and easily accessible infection [23].

Study limitations

This study was not attempting to identify definitive atypical bacterial pathogens of LRTIs like; *Chlamydia*, *Mycoplasma*, and *Legionella species* and anaerobic bacteria. In addition, Extended Spectrum Beta-lactamase (ESBL) and Carbapenemase were not detected. Moreover, this study did not categorize the distribution of bacterial LRTI pathogens as community acquired or hospital acquired.

Conclusion

This study found that more than one-third of Xpert-negative presumptive pulmonary tuberculosis adult patients were predominantly infected with other bacterial LRTI pathogens. *S. aureus* and *K. pneumoniae* were the predominant bacterial isolates, which are responsible for LRTIs. Antibiotics like meropenem and piperacillin/tazobactam are effective against Gram-negative bacteria whereas cefoxitin and gentamicin are effective against Gram-positive bacteria. However, there is a notable rise in antibiotic resistance against several clinically used antibiotics. Moreover, this study found a substantial proportion of multiple drug resistance and methicillin resistance of *S. aureus*. Primary education, history of prior antibiotic usage and crowded living conditions were significantly associated with bacterial LRTI pathogens. Therefore, routine sputum culture, antimicrobial susceptibility test and using molecular tests for better polymicrobial detection is suggested for the guidance of best therapeutic option and surveillance should be done regularly on antimicrobial resistance strains of bacterial LRTI pathogens. Promoting education, refraining from exploiting antibiotics and managing crowded living conditions would mitigate bacterial lower respiratory tract infections.

Acknowledgments

We would like to thank Haramaya University and College of Health and Medical Science for giving this opportunity to conduct this research. We would also be grateful to data collectors for their commitment and study participants for their participation in this study.

Ethical consideration

Ethical clearance was obtained from Haramaya University, College of Health and Medical Sciences through the Institutional Health Research Ethics Review Committee (IHRERC) (Ref. No. IHRERC/159/2023). A permission letter to conduct this research was obtained from Hiwot Fana Comprehensive Specialized University Hospital. Study subjects were given information about the objectives of the study and informed written consent or thumbprint for subjects who were unable to write were obtained. Subjects participated voluntarily and could withdraw from the study at any time without further consequences. Study participants who became positive for culture and antimicrobial susceptibilities were linked to antibiotic treatment regime through communicating with clinician.

Authors Disclosure

The authors declare they have no conflict of interest.

Funding

The authors received no funding for the study and publication

Author’s contributions

All authors have contributed to the work done. Negesso Gebre carried out the actual study, statistical analyses, writing the original study and manuscript write-up. Kedir Urgesa, Jemal Mohammed, Mohammed Ahmed, and Rudwan Yasin Abrahim were involved in writing the original study, developing the manuscript, supervising, validating, and all authors have agreed on the journal to submit the manuscript.

REFERENCES

1. Vos, T., et al., *Global burden of 369 diseases and injuries in 204 countries and territories, 1990–2019: a systematic analysis for the Global Burden of Disease Study 2019.* The lancet, 2020. **396**(10258): p. 1204-1222.
2. Feldman, C. and E. Shaddock, *Epidemiology of lower respiratory tract infections in adults.* Expert Review of Respiratory Medicine, 2019. **13**(1): p. 63-77.
3. Safiri, S., et al., *Global burden of lower respiratory infections during the last three decades.* Frontiers in public health, 2023. **10**: p. 1028525.
4. Yigezu, A., et al., *Burden of lower respiratory infections and associated risk factors across regions in Ethiopia: a subnational analysis of the Global Burden of Diseases 2019 study*. BMJ open, 2023. **13**(9): p. e068498.
5. Yohannes, H., et al., *Pathogenic bacteria recovered from Gene X-pert tuberculosis-negative adult patients in Gondar, Northwest Ethiopia.* BMC Pulmonary Medicine, 2023. **23**(1): p. 197.
6. Collaborators, G.L., *Estimates of the global, regional, and national morbidity, mortality, and aetiologies of lower respiratory tract infections in 195 countries: a systematic analysis for the Global Burden of Disease Study 2015*. The Lancet. Infectious diseases, 2017. **17**(11): p. 1133.
7. WHO, *WHO meeting report of a technical expert consultation: non-inferiority analysis of Xpert MTB/RIF Ultra compared to Xpert MTB/RIF*. 2017, World Health Organization.
8. Kebede, W., et al., *The value of lateral flow urine lipoarabinomannan assay and empirical treatment in Xpert MTB/RIF ultra negative patients with presumptive TB: A prospective cohort study.* Scientific Reports, 2021. **11**(1): p. 24428.
9. Baron, E.J., et al., *A guide to utilization of the microbiology laboratory for diagnosis of infectious diseases: 2013 recommendations by the Infectious Diseases Society of America (IDSA) and the American Society for Microbiology (ASM) a.* Clinical infectious diseases, 2013. **57**(4): p. e22-e121.
10. Manalan, K., J. Barrett, and O.M. Kon, *Tuberculosis clinical presentation and differential diagnosis*, in *Essential Tuberculosis*. 2021, Springer. p. 79-85.

11. Ahmed, S.K., et al., *Antimicrobial resistance: impacts, challenges, and future prospects.* Journal of Medicine, Surgery, and Public Health, 2024. **2**: p. 100081.
12. Adhikari, S., et al., *Prevalence of β-lactamase producing carbapenem-resistant enterobacteriaceae among the patients attending Bharatpur Hospital.* Biosci Discov, 2019. **10**: p. 64-71.
13. Bertrand, X. and D. Hocquet, *Antibiotic drug resistance: Causes and solutions.* EJHP Pract, 2011. **17**: p. 58-59.
14. Santella, B., et al., *Lower respiratory tract pathogens and their antimicrobial susceptibility pattern: a 5-year study.* Antibiotics, 2021. **10**(7): p. 851.
15. Troeger, C., et al., *Estimates of the global, regional, and national morbidity, mortality, and aetiologies of lower respiratory infections in 195 countries, 1990–2016: a systematic analysis for the Global Burden of Disease Study 2016.* The Lancet infectious diseases, 2018. **18**(11): p. 1191-1210.
16. FMoH, E., *Guidelines for management of TB, DR-TB and leprosy in Ethiopia.* Addis Ababa, Ethiopia Federal Ministry of Health Ethiopia, 2018.
17. Buchera, F.S., et al., *Predominance of Other Pathogenic Bacteria among Presumptive Tuberculosis Cases Attending Tuberculosis Clinics in Mwanza, Tanzania: A Cross-Sectional Laboratory-Based Study.* Microorganisms, 2022. **10**(4): p. 703.
18. Ferreira, R.L., et al., *High prevalence of multidrug-resistant Klebsiella pneumoniae harboring several virulence and β-lactamase encoding genes in a Brazilian intensive care unit.* Frontiers in microbiology, 2019. **9**: p. 3198.
19. Kebede, W., et al., *Bacterial pathogens in Xpert MTB/RIF Ultra-negative sputum samples of patients with presumptive tuberculosis in a high TB burden setting: a 16S rRNA analysis.* Microbiology Spectrum, 2024. **12**(2): p. e02931-23.
20. Gebre, A.B., T.A. Begashaw, and M.D. Ormago, *Bacterial profile and drug susceptibility among adult patients with community acquired lower respiratory tract infection at tertiary hospital, Southern Ethiopia.* BMC Infectious Diseases, 2021. **21**(1): p. 440.
21. Nurahmed, N., et al., *Bacterial profile and antimicrobial susceptibility patterns of lower respiratory tract infection among patients attending selected health centers of*

Addis Ababa, Ethiopia. The Egyptian Journal of Chest Diseases and Tuberculosis, 2020. **69**(2): p. 399-406.

22. Temesgen, D., et al., *Bacteriology of community acquired pneumonia in adult patients at Felege Hiwot Referral Hospital, Northwest Ethiopia: a cross-sectional study.* Antimicrobial Resistance & Infection Control, 2019. **8**(1): p. 101.
23. WHO, *WHO Housing and Health Guidelines.* 2018.
24. Assefa, M., et al., *Bacterial profile, antimicrobial susceptibility patterns, and associated factors of community-acquired pneumonia among adult patients in Gondar, Northwest Ethiopia: A cross-sectional study.* PloS one, 2022. **17**(2): p. e0262956.
25. Regmi, R.S., et al., *Bacterial etiology of sputum from tuberculosis suspected patients and antibiogram of the isolates.* BMC research notes, 2020. **13**: p. 1-6.
26. Cheesbrough, M.C., *District Laboratory Practice in Tropical Countries,* . Vol. Patr 2. 2006.: Cambridge, UK Cambridge University Press; .
27. CLSI, *Performance standards for antimicrobial susceptibility testing, M100, 32nd. Clinical and Laboratory Standards Institute. Wayne, PA*. 2022.
28. Rafailidis, P.I. and D. Kofteridis, *Proposed amendments regarding the definitions of multidrug-resistant and extensively drug-resistant bacteria.* Expert review of anti-infective therapy, 2022. **20**(2): p. 139-146.
29. Tchatchouang, S., et al., *Bacterial aetiologies of lower respiratory tract infections among adults in Yaoundé, Cameroon.* BioMed research international, 2019. **2019**(1): p. 4834396.
30. Attia, E.F., et al., *Tuberculosis and other bacterial co-infection in Cambodia: a single center retrospective cross-sectional study.* BMC pulmonary medicine, 2019. **19**: p. 1-7.
31. Ndiabamoh, C., *SPUTUM MICROBIAL PROFILE AND CLINICAL FEATURES OF PATIENTS WITH NEGATIVE GENEXPERT AND AFB IN MANILA, PHILIPPINES: A PILOT RETROSPECTIVE ANALYTICAL STUDY.* Chest, 2022. **161**(6): p. A146.
32. Tang, C.Y., et al., *Application of 16S rRNA metagenomics to analyze bacterial communities at a respiratory care centre in Taiwan.* Applied microbiology and biotechnology, 2015. **99**: p. 2871-2881.
33. Torres, A., et al., *Pneumonia Nat Rev Dis Primers, 7 (2021).* View in Scopus, 2021: p. 25.

34. Md, I.A. and F. Pappoe, *Prevalence of bacterial pathogens isolated from sputum cultures of hospitalized adult patients with community-acquired pneumonia at the cape coast teaching hospital, Ghana.* 2014.
35. Hassanzadeh, S., et al., *Bacterial profile and their antimicrobial resistance patterns among patients with community-acquired pneumonia in southwestern Iran.* Iranian Journal of Microbiology, 2023. **15**(3): p. 343.
36. Luan, Y., et al., *Pathogenic bacterial profile and drug resistance analysis of community-acquired pneumonia in older outpatients with fever.* Journal of International Medical Research, 2018. **46**(11): p. 4596-4604.
37. Kim, J.Y., et al., *Gram-negative bacteria's outer membrane vesicles.* Infection & Chemotherapy, 2023. **55**(1): p. 1.
38. Olugbue, V. and S. Onuoha, *Prevalence and antibiotic sensitivity of bacterial agents involved in lower respiratory tract infections.* International journal of biological and chemical sciences, 2011. **5**(2).
39. Regasa, B., et al., *Antimicrobial susceptibility pattern of bacterial isolates from community-acquired pneumonia patients in Jimma University specialized hospital, Jimma, Ethiopia.* Saudi Journal for Health Sciences, 2015. **4**(1): p. 59.
40. Khawaja, A., et al., *Etiology and outcome of severe community acquired pneumonia in immunocompetent adults.* BMC infectious diseases, 2013. **13**: p. 1-6.
41. Parker, D. and A. Prince. *Immunopathogenesis of Staphylococcus aureus pulmonary infection*. in *Seminars in immunopathology*. 2012. Springer.
42. Bharadwaj, A., et al., *Multidrug-resistant bacteria: their mechanism of action and prophylaxis.* BioMed research international, 2022. **2022**(1): p. 5419874.
43. Breijyeh, Z., B. Jubeh, and R. Karaman, *Resistance of gram-negative bacteria to current antibacterial agents and approaches to resolve it.* Molecules, 2020. **25**(6): p. 1340.
44. Alghamdi, B.A., et al., *Antimicrobial resistance in methicillin-resistant Staphylococcus aureus.* Saudi journal of biological sciences, 2023. **30**(4): p. 103604.
45. González-Vázquez, R., et al., *Detection of mecA Genes in Hospital-Acquired MRSA and SOSA Strains Associated with Biofilm Formation.* Pathogens, 2024. **13**(3): p. 212.

46. Dejene, S., et al., *Bacterial Pathogens, Antimicrobial susceptibility Pattern and predictors of Lower Respiratory Tract Infection among Children admitted in health care settings in South Ethiopia.* 2023.
47. Liu, J., et al., *A causal relationship between educational attainment and risk of infectious diseases: A Mendelian randomisation study.* Journal of Global Health, 2024. **14**.
48. Alvis-Zakzuk, N.J., et al., *Education and pneumonia mortality: a trend analysis of its inequalities in Colombian adults.* BMJ open respiratory research, 2020. **7**(1): p. e000695.
49. Gao, B., et al., *Molecular epidemiology and risk factors of ventilator-associated pneumonia infection caused by carbapenem-resistant enterobacteriaceae.* Frontiers in Pharmacology, 2019. **10**: p. 262.
50. Gross, A.E., et al., *Epidemiology and predictors of multidrug-resistant community-acquired and health care-associated pneumonia.* Antimicrobial agents and chemotherapy, 2014. **58**(9): p. 5262-5268.
51. Mancuso, G., et al., *Bacterial antibiotic resistance: the most critical pathogens.* Pathogens, 2021. **10**(10): p. 1310.
52. Temesgen, D., et al., *Bacteriology of community acquired pneumonia in adult patients at Felege Hiwot Referral Hospital, Northwest Ethiopia: a cross-sectional study.* Antimicrobial Resistance & Infection Control, 2019. **8**: p. 1-8.

Table 1: Socio-demographic characteristics of study participants at HFCSH, Harar, eastern, Ethiopia , 2024 (n=371)

**Variables**	**Category**	**Frequency**	**Percentage (%**
Sex	Male	197	53.1
	Female	174	46.9
Age	18-35	184	49.6
	36-49	94	25.3
	50-64	66	17.8
	Above 65	27	7.3
Marital status	Unmarried	119	32.1
	Married	241	65.0
	Divorced/separated	5	1.3
	Widowed	6	1.6
Residence	Urban	158	42.6
	Rural	213	57.4
Educational status	No formal Education	124	33.4
	Primary	146	39.4
	Secondary	58	15.6
	College and above	43	11.6
Occupation	Farmer	127	34.2
	Factory	14	3.8
	Government	49	13.2
	Private	27	7.3
	House wife	68	18.3
	Student	78	21.0
	Others	8	2.2
Family size	2-4	145	39.1
	5-7	185	49.9
	Above 8	41	11.1
Patient category	Inpatient	13	3.5
	Outpatient	358	96.5

Table 2: Clinical history, behavioral, and environmental characteristics of the study participants at HFCSH, Harar, eastern Ethiopia, 2024 (n=371)

**Variables**	**Category**	**Frequency**	**Percentage (%)**
Comorbidity	Diabetes	12	3.2
	HIV	8	2.2
	Heart disease	7	1.9
	Asthma	7	1.9
History of hospital admission	Yes	40	10.8
	No	331	89.2
History of Tuberculosis	Yes	17	4.6
	No	354	95.4
History of antibiotic usage prior a month and above	Yes	192	51.8
	No	179	48.2
Smoking cigarettes	Yes	113	30.5
	No	258	69.5
Alcohol use	Never	291	78.4
	Sometimes	10	2.7
	Always	70	18.9
Chewing khat	Yes	188	50.7
	No	183	49.3
Hand washing after outing	Never	31	8.4
	Sometimes	307	82.7
	Always	33	8.9
Crowded living condition	Yes	155	41.8
	No	216	58.2
Separate kitchen from house	Yes	335	90.3
	No	36	9.7
Source of energy for cooking	firewood	226	60.9
	Electric power	145	39.1
Living in mud/soil floor	Yes	187	50.4
	No	184	49.6
Exposed to dust at work	Yes	231	62.3
	No	140	37.7
Source of drinking water	Improved	151	40.7
	Unimproved	220	59.3

Table 3: Antimicrobial susceptibility patterns of Gram-positive bacterial isolates from sputum culture of adult patients with Xpert -negative presumptive tuberculosis at HFCSH, eastern Ethiopia, 2024 (N=42)

Bacterial isolates	Total n (%)	S R (%)	Antimicrobial agents								
			P	FOX	CD	AZT	E	SXT	TTC	CIP	CN
S. aureus	24 (19)	S<br>R	5(20.8)<br>19(79.2)	20(83.3)<br>4(16.7)	16(66.7)<br>8(33.3)	18(75)<br>6(25)	17(70.8)<br>7(29.2)	14(58.3)<br>10(41.7)	5(20.8)<br>19(79.2)	20(83.3)<br>4(16.7)	20(83.3)<br>4(16.7)
S. pemoniae	13 (10.3)	S<br>R	NA	NA	10(76.9)<br>3(23.1)	NA	6(46.2)<br>7(53.8)	8(61.5)<br>5(38.5)	6(46.2)<br>7(53.8)	NA	NA
S. pypgens	5 (4)	S<br>R	NA	NA	3(60)<br>2(40)	NA	2(40)<br>3(60)	NA	0<br>5(100)	NA	NA
Total	42 (33.3)	S<br>R	5(20.8)<br>19(79.2)	20(83.3)<br>4(16.7)	29(69)<br>13(31)	18(75)<br>6(25)	25(59.5)<br>17(40.5)	22(59.5)<br>15(40.5)	11(26.2)<br>31(73.8)	20(83.3)<br>4(16.7)	20(83.3)<br>4(16.7)

Note: S=Sensitive, R= Resistant, NA= Not applicable, P=Penicillin, FOX=Cefoxitin, CD=Clindamycin, AZT=Azithromycin, E=Erythromycin, SXT= trimethoprim–sulfamethoxazole, TTC=Tetracycline, CIP= Ciprofloxacin, CN=Gentamycin

Table 4: Antimicrobial susceptibility patterns of Gram-negative bacterial isolates from sputum culture of adult patients with Xpert MTB/RIF-negative presumptive tuberculosis at HFCSH, eastern Ethiopia,2024 (N=84)

Bacterial species N (%)	S R (%)	Antimicrobial agents								
		AMP	CN	AUG	CRO	SXT	CAZ	CIP	TZP	MEM
K. pneumonia	S	NA	11(61.1)	6(33.3)	15(83.3)	9(50)	15(83.3)	15(83.3)	18(100)	18(100)
18(14.3)	R		7(38.9)	12(66.7)	3(16.7)	9(50)	3(16.7)	3(16.7)	0	0
K. oxytoca	S	NA	8(80)	3(30)	6(60)	7(70)	7(70)	9(90)	10(100)	9(90)
10(7.9)	R		2(20)	7(70)	4(40)	3(10)	3(10)	1(10)	0	1(10)
	S	NA	7(77.8)	4(44.4)	5(55.6)	5(55.6)	6(66.7)	7(77.8)	8(88.9)	8(88.9)
K.ozanae 9(7.1)	R		2(22.2)	5(55.6)	4(44.4)	4(44.4)	3(33.3)	2(22.2)	1(11.1)	1(11.1)
K.rhinoscleromatis	S	NA	8(61.5)	6(46.2)	9(69.2)	6(46.2)	8(61.5)	8(61.5)	11(84.6)	13(100)
13(10.3)	R		5(38.5)	7(53.8)	4(30.8)	7(53.8)	5(38.5)	5(38.5)	2(15.2)	0
E. coli	S	1(12.5)	7(87.5)	3(37.5)	5(62.5)	5(62.5)	5(62.5)	4(50)	8(100)	8(100)
8(6.3)	R	7(88.5)	1(12.5)	5(62.5)	3(37.5)	3(37.5)	3(37.5)	4(50)	0	0
Enterobacter spp.	S	NA	4(57.1)	4(57.1)	5(71.4)	4(57.1)	7(100)	6(85.7)	7(100)	7(100)
7(5.6)	R		3(42.9)	3(42.9)	2(28.6)	3 (42.9)	0	1(14.3)	0	0
Citrobacter spp.	S	NA	4(100)	NA	3(75)	3(75)	1(25)	4(100)	4(100)	4(100)
4(3.2)	R		0		1(25)	1(25)	3(75)	0	0	0
P.mirabilis	S	1(100)	1(100)	1(100)	1(100)	1(100)	1(100)	1(100)	1(100)	1(100)
1(0.8)	R	0	0	0	0	0	0	0	0	0
H. influanzae	S	0	NA	6(100)	5(83.3)	5(83.3)	NA	6(100)	6(100)	6(100)
6(4.8)	R	6(100)		0	1(16.7)	1(16.7)		0	0	0
M. catarrhalis	S	4(50)	5(62.5)	4(50)	6(75)	1(12.5)	8(100)	6(75)	8(100)	8(100)
8(6.3)	R	4(50)	3(37.5)	4(50)	2(25)	7(87.5)	0	2(25)	0	0
Total 84(66.7)	S	6(26.1)	55(70.5)	37(46.3)	60(71.4)	46(54.8)	58(74.4)	66(78.6)	81(96.4)	82(97.6)
	R	17(73.9)	23(29.5)	43(53.7)	24(28.6)	38(45.2)	20(25.6)	18(21.4)	3(3.6)	2(2.4)

Note: S=Sensitive, R= Resistant, NA=Not applicable, AMP=Ampicillin, CN=Gentamycin, AUG=Augmentin, CRO=Ceftriaxone, SXT= trimethoprim–sulfamethoxazole, CAZ=Ceftazidime, CIP=Ciprofloxacin, TZP=Pipracillin/Tazobactem, MEM=Meropenem

Table 5: Multi-drug resistance patterns of bacterial isolates from sputum culture of adult patients with Xpert-negative presumptive tuberculosis at HFCSH, eastern Ethiopia, 2024 (N= 50)

Bacterial isolates	N (%)	Resistance pattern (%)						
		RO	R1	R2	R3	R4	≥ R5	MDR
S. aureus	24(19)	-	-	-	-	3(12.5)	6(25)	9(37.5)
S. pemoniae	13(10.3)	-	-	-	1(7.7)	3(23.1)	-	4(30.7)
S. pypgens	5(4)	-	-	1(20)	1(20)	-	1(20)	2(40)
Gram-positive	42(33.3)	-	-	1(2.4)	2(4.7)	6(14.3)	7(16.7)	15(35.7)
K. pneumonia	18(14.3)	2(11.1)	-	-	3(16.7)	-	3(16.7)	6(33.3)
K. oxytoca	10(7.9)	1(10)	2(20)	1(10)	2(20)	1(10)	1(10)	4(40)
K.ozanae	*9(7)*	-	2(22.2)	2(22.2)	1(11.1)	2(22.2)	1(11.1)	4(44.4)
K.rhinoscleromatis	*13(10.3)*	1(7.7)	-	1(7.7)	-	1(7.7)	5(38.5)	6(46.2)
E. coli	8(6.3)	2(25)	1(12.5)	-	3(37.5)	1(12.5)	2(25)	6(75)
Enterobacter species	7(5.6)	3(42.8)	1(14.3)	1(14.3)	3(42.8)	-	-	3(42.8)
Citrobacter Species	4(3.2)	4(100)	2(50)	-	1(25)	-	-	1(25)
Proteus mirabilis	1(0.8)	1(100)	-	-	-	-	-	-
H. influenza	6(4.8)	4(66.7)	2(33.3)	-	-	-	1(16.7)	1(16.7)
M. catarrhalis	8(6.3)	3(37.5)	-	2(25)	1(12.5)	2(25)	1(12.5)	4(50)
Gram-negative	84(66.7)	21(25)	10(11.9)	7(8.3)	14(16.7)	7(8.3)	14(16.7)	35(41.7)
Total	126(100)	21(16.7)	10(7.9)	8(6.3)	16(12.6)	13(10.3)	21(16.7)	50(39.6)

Note: R0, no antibiotic resistance to all antibiotics ; R1, resistance to one antibiotic; R2, resistance to two antibiotics ; R3, resistance to three antibiotics; R4, resistance to four antibiotics; ≥R5, resistance to five and more antibiotics

Table 6: Bi-variable and multivariable logistic regression analyses of factors associated with bacterial LRTI pathogens among Xpert-negative presumptive tuberculosis adult patients at HFCSH, eastern Ethiopia, 2024

**Variables**	**Culture result**		**Bi-variable analysis**		**Multivariable analysis**	
	**Positive n (%)**	**Negative n (%)**	**COR (95% CI)**	***p* -value**	**AOR (95% CI))**	***p* -value**
Gender						
Male	60(30.5)	137(69.5)	1			
female	66(37.9)	108(62.1)	1.395(.907, 2.151)	0.130*	1.482(0.894,2.458)	0.128
Age						
18-35	63(34.2)	121(65.8)	1			
36-49	28(29.8)	66(70.2)	0.815(0.472,1.385)	0.455		
50-64	23(34.8)	43(65.2)	1.027(0.563,1.843)	0.929		
≥ 65	12(44.4)	15(55.6)	1.537(0.667, 3.477)	0.303		
Marital status						
Unmarried	39(32.8)	80(67.2)	0.975(0.182,7.248)	0.977		
Married	84(34.9)	157(65.1)	1.070(0.205,7.830)	0.938		
Divorce	1(20)	4(80)	0.500(0.018,7.537)	0.624		
Widowed	2(33.3)	4(66.7)	1		.	
Residence						
Urban	53(33.5)	105(66.5)	1			
Rural	73(34.3)	140(65.7)	1.033(0.669,1.599)	0.884		
Educational level						
No informal	45(36.3)	79(63.7)	0.871(0.429,1.797)	0.704		
Primary	44(30.1)	102(69.9)	0.660(0.327,1.352)	0.248*	4.008(1.619,9.920)	0.003**
Secondary	20(34.5)	38(65.5)	0.805(0.355,1.829)	0.603		
College and above	17(39.5)	26(60.5)	1			
Occupational status						
Farmer	35(27.6)	92(72.4)	0.634(0.148,3.220)	0.547		
Factory	5(35.7)	9(64.3)	0.926(0.152,6.088)	0.933		
Government	18(36.7)	31(63.3)	0.968(0.211,5.163)	0.967		
Private	11(40.7)	16(59.3)	1.146(0.230,6.527)	0.870		
House wife	27(39.7)	41(60.3)	1.098(0.248,5.703)	0.904		
Student	27(34.6)	51(65.4)	0.882(0.201,4.562)	0.871		
Other	3(37.5)	5(62.5)	1			
Family size						
2-4	45(31)	100(69)	1			
5-7	67(36.2)	118(63.8)	1.262(0.796,2.010)	0.324		
Above 8	14(34.1)	27(65.9)	1.152(0.542,2.377)	0.706		
Alcohol use						
Never	94(32.3)	197(67.7)	1			
Sometimes	5(50)	5(50)	2.096(0.570,7.704)	0.251		
Always	27(38.6)	43(61.4)	1.316(0.760,2.249)	0.319		

Smoking						
Yes	43(38.1)	70(61.9)	1.295(0.814,2.050)	0.271		
No	83(32.2)	175(67.8)	1			
Chewing khat						
Yes	62(33)	126(67)	0.915(0.595,1.407)	0.685		
No	64(35)	119(65)	1			
Comorbidity						
DM	5(41.7)	7(58.3)	1.786(0.255,16.23)	0.570		
HIV	3(37.5)	5(62.5)	1.500(0.170,15.55)	0.715		
HD	2(28.6)	5(71.4)	1.00(0.088,11.318)	1.000		
Asthma	2(28.6)	5(71.4)	1			
Admission history						
Yes	19(47.5)	21(52.5)	1.894(0.971,3.678)	0.059*	1.074(0.497,2.321)	0.856
No	107(32.3)	224(67.7)	1			
History of TB						
Yes	6(35.3)	11(64.7)	1.064(0.359,2.866)	0.906		
No	120(33.9)	234(66.1)	1			
Prior antibiotic usage						
Yes	81(42.2)	111(57.8)	2.173(1.401, 3.401)	0.001*	1.866(1.132,3.077)	0.014**
No	45(25.1)	134(74.9)	1			
Hand washing						
Never	15(48.4)	16(51.6)	0.996 (0.372,2.669)	0.994		
Sometimes	95(30.9)	212(69.1)	0.476(0.230, 0.990)	0.045*	1.046(0.319,3.424)	0.941
Always	16(48.5)	17(51.5)	1			
Crowded living						
Yes	86(55.5)	69(44.5)	5.484(3.461, 8.822)	0.000*	5.106 (3.028, 8.611)	0.000**
No	40(18.5)	176(81.5)	1			
Separate kitchen						
yes	119(35.5)	216(64.5)	1			
No	7(19.4)	29(80.6)	0.438(0.172,0.977)	0.059*	0.460(0.174,1.217)	0.118
Living in mud floor						
Yes	56(29.9)	131(70.1)	0.696(0.451,1.071)	0.100*	0.803(0.45,1.432)	0.457
No	70(38)	114(62)	1			
Cooking energy						
Firewood	75(33.2)	151(66.8)	0.915(0.591,1.424)	0.693		
Electric	51(35.2)	94(64.8)	1			
Dust exposure						
Yes	75(32.5)	156(67.5)	0.839(0.540,1.307)	0.435		
No	51(36.4)	89(63.6)	1			
Drink water source						
Unimproved	66(30)	154(70)	0.650(0.420,1.005)	0.052*	0.828(0.493,1.389)	0.474
Improved	60(39.7)	91(60.3)	1			

Note: * Statistically significant at *p* <0.25, in bi-variable analysis, ** statistically at *p* <0.05, in multi-variable analysis, AOR= Adjusted odd ratio, COR= Crude odd ratio, CI= Confidence interval

Click here to access/download
Supporting Information
Waiver_1 Dec 2025.doc
