# Supplementary material for: Uncovering overlooked bacterial infections in gene X-pert MTB/RIF negative sputum samples of adult patients with presumptive tuberculosis in Harar, Eastern Ethiopia
Source: PLoS One. 2026 May 14;21(5):e0348978. doi: 10.1371/journal.pone.0348978 (PMC13175332; doi:10.1371/journal.pone.0348978)
Supplement: Attachment — Submitted filename: Response to Reviewer.docx [file pone.0348978.s004.docx]

| **Comments to the Author** | Authors response |
| --- | --- |
| 1. Is the manuscript technically sound, and do the data support the conclusions?  The manuscript must describe a technically sound piece of scientific research with data that supports the conclusions. Experiments must have been conducted rigorously, with appropriate controls, replication, and sample sizes. The conclusions must be drawn appropriately based on the data presented.  Reviewer #1: Yes  Reviewer #2: Yes | Thank you so much dear reviewer.  The comments, suggestions and recommendation you have given us helping us to enrich our revised manuscript and we have considered and corrected all the points on your concern in both clean revised manuscript and track changed manuscript. |
| 2. Has the statistical analysis been performed appropriately and rigorously?  Reviewer #1: Yes  Reviewer #2: N/A | Thank you for suggestions.  The authors have performed the statistical analysis using SPSS. Then, frequency distribution, percentages and binary logistic regression were presented in manuscript. We have calculated sample size, presented p-values, odd ratio and confidence intervals to indicate the statistical significance of factors associated with diseases. Finally, the multivariable logistic regression analysis were used for final decisions with odds ratio and 95% confidence intervals. Statistical significance was declared at a p-value < 0.05. In this way we have performed relevant statistical analysis that could be tracked in the manuscript. We are thankful for your comments and suggestions. |
| 3. Have the authors made all data underlying the findings in their manuscript fully available? The [PLOS Data policy](http://track.editorialmanager.com/CL0/http:%2F%2Fwww.plosone.org%2Fstatic%2Fpolicies.action%23sharing/1/010f019c039aa0f4-4916a6e3-180f-4f4d-b45a-a41386b315ce-000000/l1iAeBy8TS2tQ8pjADaCP4mPaSJK0sDI12P4G5yRZ-E=248) requires authors to make all data underlying the findings described in their manuscript fully available without restriction, with rare exception (please refer to the Data Availability Statement in the manuscript PDF file). The data should be provided as part of the manuscript or its supporting information, or deposited to a public repository. For example, in addition to summary statistics, the data points behind means, medians and variance measures should be available. If there are restrictions on publicly sharing data—e.g. participant privacy or use of data from a third party—those must be specified.  Reviewer #1: No  Reviewer #2: Yes | Thank you so much.  Data regarding the finding is available in the manuscript, the authors have done and present most of the statics in the manuscript. There is no restriction on accessing data, and we hae included raw data supporting the study as aa supplementary file. |
| 4. Is the manuscript presented in an intelligible fashion and written in standard English?  PLOS ONE does not copyedit accepted manuscripts, so the language in submitted articles must be clear, correct, and unambiguous. Any typographical or grammatical errors should be corrected at revision, so please note any specific errors here.  Reviewer #1: Yes  Reviewer #2: No | We are thanks all the reviewer for the constructive comments and suggestions.  Accordingly, the authors have taken time, revised and corrected all typographical error, language clarity and any an unambiguous words.  Therefore, the reviewer can easily track the correction in the clean revised manuscript and track changed manuscript. |
| **Reviewer comments** | **Author Response to Reviewer** |
| **Introduction** Page 11, Lines 92-95: The authors state that studies on this topic are "rare".  To ensure scientific precision, please clarify if 'rare' refers to a national context (Ethiopia) or specifically to the Eastern region (Harar). The authors are encouraged to acknowledge and cite relevant existing work to better contextualize their study. For example - a) Yohannes, Hana, et al. "Pathogenic bacteria recovered from Gene X-pert tuberculosis-negative adult patients in Gondar, Northwest Ethiopia." BMC pulmonary medicine 23.1 (2023): 197. b) Kebede, Wakjira, et al. "Bacterial pathogens in Xpert MTB/RIF Ultra-negative sputum samples of patients with presumptive tuberculosis in a high TB burden setting: a 16S rRNA analysis." Microbiology Spectrum 12.2 (2024): e02931-23. | Thank you so much for the constructive comments.  In this study when we said rare data, it’s in eastern parts of Ethiopia where there is no published study or data on Gene Xpert negative presumptive TB patients. We have also cited available source in including that recommended by reviewer as it is relevant to this study.  Accordingly, the authors have made correction in both clean revised and tracked changed manuscript. |
| **Methods and Results** Figure 1 missing (Page 16, Line 234): The manuscript mentions "Figure 1," but this figure appears to be missing from the submitted files and supplementary data. Please provide this figure for review. | Thank you dear reviewer for your constructive comments.  The authors have accepted your comments, and suggestions. Accordingly, we mistakenly missed Fig 1 during submission, however now we have incorporated into the main manuscript following the Fig 1 citation in both clean revised manuscript and tracked changed manuscript. |
| To improve the clarity and impact of the results, the authors are encouraged to convert some of the dense tabular data into figure/plots, while keeping the raw data in table/supplementary table: 1) Table 6: This table would be better represented as a Forest Plot. Visualizing the Adjusted Odds Ratios (AOR) and 95% Confidence Intervals would allow for an immediate comparison of the significance of risk factors like crowding and education. | The authors have included two figures as per the reviewer suggestions, and clarify other tables so that it could be easy to understand for the reader.  Our esteemed reviewers can easily check the revision in both clean revised manuscript and track changed manuscript. |
| 2) Tables 3 & 4: The antimicrobial susceptibility data is quite dense. The authors should consider using grouped or stacked bar charts to represent resistance patterns for the most prevalent isolates (e.g., S. aureus and K. pneumoniae). This would clearly highlight the high resistance to first-line drugs like Ampicillin versus the susceptibility to Meropenem. | The authors have added two figures indicating S*. aureus and K. pneumonia antimicrobial* susceptibility profile on available antibiotic at the hospital separately in the document next to Figure citation that highlight bacterial antibiotic profile. |
| **Discussion** The study found that 34% of patients had a bacterial infection. This means 66% of the symptomatic patients remained undiagnosed. The authors should expand the discussion to address this "gap." Is it possible that viral pathogens, fastidious bacteria not captured by standard culture, or Non-Tuberculous Mycobacteria (NTM) are responsible for these symptoms? | The authors thanks the reviewer for invaluable comments and suggestion that help us to enrich the revision of the manuscript.  In fact LRTIs are not only caused by cultivable bacteria, but also by fastidious bacteria not captured by standard culture, anaerobic bacteria and virus. We have addressed this issue in discussion parts and limitations sections of study. |
| Typically, S. pneumoniae is the leading cause of community-acquired LRTIs. However, this study found S. aureus (19%) to be the most common in the tested participants. The authors should discuss why Harar might have such high S. aureus levels. | Thank you very much dear reviewer for the comments.  As mentioned by the authors in the study's limitations section, this research did not differentiate between community-acquired and hospital-acquired infections. Instead, it concentrated on identifying bacterial lower respiratory tract infections based on Gene-Xpert results, which is why it was challenging to identify *S. aureus and S. pneumoniae.* So, this figure is overall bacterial LRTIs not based on community acquired or hospital acquired. Thank you so much for your understanding. |
| **Limitations**  The authors should acknowledge in the limitations section that the reliance on manual biochemical identification rather than molecular methods (such as PCR or 16S rRNA sequencing) likely resulted in an underestimation of the true bacterial burden, particularly for fastidious or non-culturable organisms. | The authors are thankful to reviewer for their constructive recommendations  The authors have addressed these points because conventional culture media and biochemical methods were insufficient for detecting certain organisms responsible for lower respiratory tract infections (LRTIs). Specifically, they were unable to identify bacteria such as Chlamydia, Mycoplasma, Legionella, and, to a lesser extent, anaerobic bacteria, as well as viral pathogens associated with LRTIs. The authors have recognized and clarified this issue in the limitations section of the study. |
| Reviewer #2: This articles needs to be modified according to the suggestions made in the comments of the manuscript. The manuscript does not mention about the study being approved by the institutional research committee along with approval number to be mentioned. Materials and methods some of the information are added which are not necessary to be included in the manuscript Conclusion must be written briefly | Dear esteemed reviewer, thank you so much for your comments.  The authors mentioned Ethical approval and provided approval number under Ethical consideration in main manuscript as indicated below “Ethical clearance was obtained from Haramaya University, College of Health and Medical Sciences through the Institutional Health Research Ethics Review Committee (IHRERC) (Ref. No. IHRERC/159/2023)”.  The method part are revised and unnecessary information was removed. Conclusion part are briefly rewritten in revised manuscript based on the finding of the study in both clean and tracked changed revised manuscript. |
